# Application of pulsed laser ablation (PLA) for the size reduction of non-steroidal anti-inflammatory drugs (NSAIDs)

**DOI:** 10.1038/s41598-020-72865-z

**Published:** 2020-09-25

**Authors:** Tamás Gera, Eszter Nagy, Tamás Smausz, Judit Budai, Tibor Ajtai, Fruzsina Kun-Szabó, Zsolt Homik, Judit Kopniczky, Zoltán Bozóki, Piroska Szabó-Révész, Rita Ambrus, Béla Hopp

**Affiliations:** 1 Department of Optics and Quantum Electronics, University of Szeged, Dóm tér 9, 6720 Szeged, Hungary; 2 Department of Materials Science, Interdisciplinary Excellence Centre, University of Szeged, Dugonics tér 13, 6720 Szeged, Hungary; 3 Institute of Pharmaceutical Technology and Regulatory Affairs, University of Szeged, Eötvös utca 6, 6720 Szeged, Hungary

**Keywords:** Applied physics, Lasers, LEDs and light sources, Drug delivery

## Abstract

We studied the application of pulsed laser ablation (PLA) for particle size reduction in non-steroidal anti-inflammatory drugs (NSAIDs). Grinding of the poorly water-soluble NSAID crystallites can considerably increase their solubility and bioavailability, thereby the necessary doses can be reduced significantly. We used tablets of ibuprofen, niflumic acid and meloxicam as targets. Nanosecond laser pulses were applied at various wavelengths (KrF excimer laser, λ = 248 nm, FWHM = 18 ns and Nd:YAG laser, λ_1_ = 532 nm/λ_2_ = 1064 nm, FWHM = 6 ns) and at various fluences. FTIR and Raman spectra showed that the chemical compositions of the drugs had not changed during ablation at 532 nm and 1064 nm laser wavelengths. The size distribution of the ablated products was established using two types of particle size analyzers (SMPS and OPC) having complementary measuring ranges. The mean size of the drug crystallites decreased from the initial 30–80 µm to the submicron to nanometer range. For a better understanding of the ablation mechanism we made several investigations (SEM, Ellipsometry, Fast photography) and some model calculations. We have established that PLA offers a chemical-free and simple method for the size reduction of poorly water-soluble drugs and a possible new way for pharmaceutical drug preformulation for nasal administration.

## Introduction

Many widely applied non-steroidal anti-inflammatory drugs (NSAIDs) are poorly water-soluble and have relatively low bioavailability. Consequently, improving their solubility and dissolution rate would provide a great opportunity to lower the required doses and reduce the risk of side effects. To address these challenges research teams have developed several techniques such as crystal engineering, salt formation, solid dispersion, amorphization and particle size reduction, but these procedures often require complex laboratory work^1–6^.

Particle size reduction is one of the most popular and efficient methods to produce micro- or nanoparticles in order to increase the specific surface area and thereby improve the dissolution rate of poorly soluble drugs. In this case the dissolution rate is directly proportional to the specific surface area, therefore the dissolution rate, the saturation concentration and the bioavailability of the drugs can all be enhanced^7^. The two conventional and straightforward size reduction methods are comminution (grinding, milling) and spray drying. Both methods apply mechanical stress for the disaggregation of the active compound, but the smallest attainable sizes still remain in the micrometer regime^8,9^. Advanced methods can reach the upper part of sub-micrometer ranges but the procedures involved are sometimes complicated or need additional materials, which makes it more difficult to produce particles in this size range^10–13^.

Pulsed laser ablation (PLA) is a well-known technique for nanoparticle generation in case of inorganic materials. During PLA a high-energy pulsed laser beam is focused onto the target, which results in micro-explosions close to the surface, and the ejection of particles. Ablated products are collected for further analysis^14–17^. It has already been shown that PLA/PLD is suitable for the size reduction of pharmaceutical particles without causing any chemical decomposition. In recent years, researchers have also started to use lasers for particle size reduction in drugs in liquid environment (PLAL)^18–23^.

In this work, three NSAIDs (ibuprofen, meloxicam and niflumic acid) with different chemical structures but similar solubility, dissociation constant, particle size and crystallinity were selected as targets to study the effect of a high-energy pulsed laser beam on the chemical degradation and particle size distribution of the ablated drug particles. Being selective cyclooxygenase (COX-2) inhibitors, these model drugs are used in acute pain therapy where a basic requirement is rapid absorption through the gastric mucosa^24^. Since these drugs have a weak acidic character, their solubility in gastric juice (pH = 1.2) is very poor. On the other hand, ablated particles dissolve faster and can produce rapid analgesic effect.

Our aim was to study the validity of the PLA method for particle size reduction in different drugs with low dissolution rates, in ambient gas at normal pressure. Ibuprofen, meloxicam and niflumic acid are frequently used, poorly water-soluble NSAIDs, representing three different molecular derivatives. We also wanted to explore the mechanism of PLA when drug targets with different thermal and optical properties are ablated under various experimental conditions. To cover the UV–VIS–IR regime with ablating laser wavelengths, a KrF excimer laser (λ = 248 nm) and a Nd:YAG laser system (λ_1_ = 1064 nm, λ_2_ = 532 nm) were used. We adjusted laser fluences at each wavelength to the optical and mechanical properties of the target materials. Ablated products were collected at normal ambient pressure using N_2_ gas flow. We studied the chemical composition and particle size distribution of the ablated particles, the optical absorption of the target drugs and made fast photography measurements too. Our motivation was to show that PLA, a simple and chemical-free particle grinding method, has a great potential in pharmaceutics. Laser ablation produced drug particles can be preferable during the preparation of medicines for per os, nasal and pulmonary drug administration. Pulmonary administration of anti-inflammatory drugs like meloxicam or ibuprofen can be especially significant in the treatment of pneumonia or severe acute respiratory syndrome caused by viral infections (e.g. coronavirus). It has been shown that only sub-micrometer size particles can get to the lower lung area (alveolar region)^25^. Therefore, size-reduced drug particles produced by PLA may form the basis of a new type of pulmonary drug formulation for the fast and effective treatment of the hard-to-reach alveolar region of the lung.

## Materials

### Ibuprofen

Ibuprofen (α-Methyl-4-(isobutyl) phenylacetic acid) was obtained from Sigma-Aldrich Ltd., (Saint Louis, Missouri, USA). It is a white powder with a particle size of 15.3 μm (d(0.5)). NSAID classification: Propionic acid derivatives. Thermal properties: Melting point T_m_ = 75–77 °C; Boiling point T_b_ = 157 °C; Degradation temperature T_dec_ = 230 to 250 °C.

### Meloxicam

Meloxicam(4-hydroxy-2-methyl-N-(5-methyl-2-thiazolyl)-2H-benzothiazine-3-car-boxamide-1,1-dioxide) was obtained from EGIS Ltd., (Budapest, Hungary). It is a yellow powder with a particle size of 3.78 μm (d(0.5)), 100% crystalline. NSAID classification: Enolic acid (oxicam) derivatives. Thermal properties: Melting point and decomposition temperature are the same, T_m_ = T_dec_ = 254 °C.

### Niflumic acid

Conventional niflumic acid (NIF) (2-[[3-(trifluoromethyl)phenyl] amino] -3-pyridinecarboxylic acid) was purchased from G. Richter Pharmaceutical Factory, Budapest, Hungary. The particle size is 18.85 μm (d(0.5)). NSAID classification: Anthranilic acid derivatives (fenamates). Thermal properties: Melting point and decomposition temperature are the same T_m_ = T_dec_ = 203 °C.

## Experiments

### Pulsed laser ablation (PLA) of drug tablets at different laser wavelengths

We applied three different laser wavelengths for pulsed laser ablation (PLA) of various drug tablets. For UV irradiation a KrF excimer laser (LLG Twinamp, FWHM = 18 ns, λ = 248 nm, f = 10 Hz) was used, while for VIS and IR ablation we applied the first and second harmonics of a Nd:YAG laser (Quantel, FWHM = 6 ns, λ = 532 nm/1064 nm, f = 10 Hz), respectively. We varied the number of laser pulses and adjusted the fluence from 1.5 to 12 J cm^−2^. In order to achieve a significant ablation yield, we used fluences slightly above the ablation threshold. The highest applied fluence was limited by the mechanical fracture of the target tablets. The tablets were produced from commercially available drug powders using a hydraulic compactor at 175 MPa pressure.

**Figure 1:**
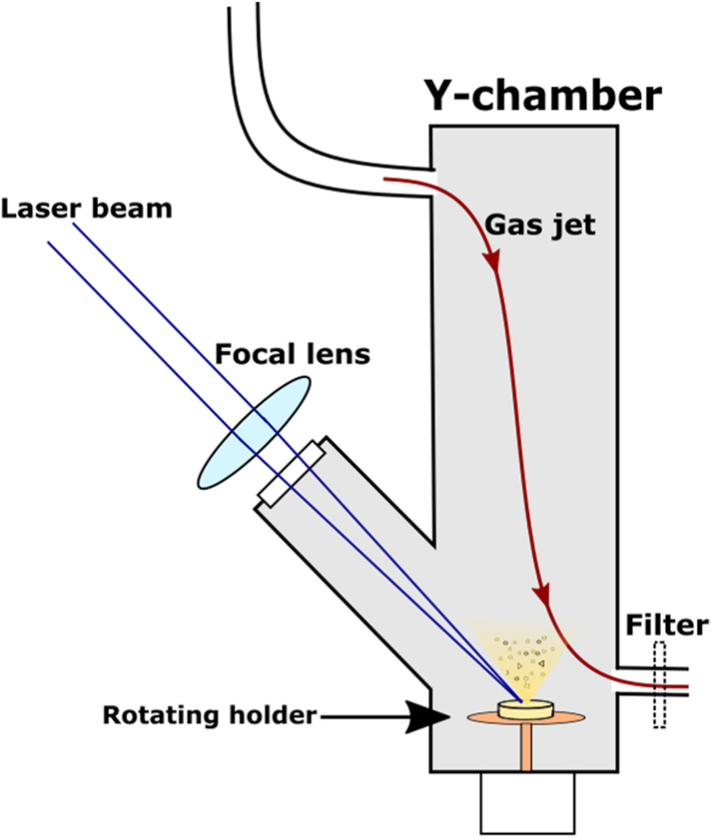
Experimental setup.

Our experimental setup is shown in Fig. 1. The tablets were placed on a rotating sample holder at the bottom of the reaction chamber. The laser beam was focused on the target surface at a grazing angle of 45° by a fused silica (UV) and an N-BK7 (VIS-IR) fused silica Plano Convex lens (focal length = 17.5 cm). To be able to direct and control the ablated particles, we created a flow field of N_2_ gas along the chamber. A constant flow rate (0.3–2 l/min) was maintained by a T-element at the entrance of the gas jet. The ablated particles were either collected for chemical analysis on a filter (pore size ~ 1 μm, Merck Millipore Ltd. Omnipore Membrane Filter), or they directly entered the particle size analyzer at the exit of the gas stream. Theoretically, the pore size of the membrane filter is 1 μm, yet smaller elements can also be collected as the pores fill up with particles reaching the filter first.

### Chemical composition characterization methods

#### Fourier transformed infrared spectroscopy (FTIR)

The preservation of the medical effect is an essential requirement during particle size reduction in pharmaceutical substances. To ensure that the chemical composition of the ablated products is identical with that of the initial drugs, the ablated particles were analyzed by FTIR spectroscopy (Thermo Nicolet AVATAR 330, LabX Midland, ON, Canada). The particles were collected from the filters and mixed with KBr powder for pellet formation. FTIR spectra were recorded in the 4000–400 cm^−1^ range, at a resolution of 4 cm^−1^, using the average of 128 scans.

#### Raman spectroscopy

Raman spectroscopy measurements were carried out by a Thermo Scientific, DXR Raman microscope. Laser beam of λ = 780 nm wavelength and 1–10 mW power was used with a 0.7 μm spot size on the sample. We applied a 25 μm slit spectrograph aperture and 900 lines/mm grating. Raman spectra were recorded in the 200–1800 cm^−1^ range with a resolution of 5.4–8.3 cm^−1^. All spectra were recorded by scanning 10 times with a 4 s integration time.

### Particle size analysis

PLA provides an efficient way for grinding; however, the size distribution of the ablated particles spans a wide range, wherefore we used two types of size analyzers, having complementary, slightly overlapping measuring ranges.

#### Scanning mobility particle sizer (SMPS)

We investigated the particle size distribution in the 10–800 nm range with SMPS (GRIMM system, Aerosol Technik, Germany, type SMPS + C). We set the scanning time to 7 min and repeated the measurements three times. The gas flow was 0.3 l/min during the scans.

#### Optical particle counter (OPC)

To establish if particles larger than 800 nm were also produced, we used an OPC (GRIMM system, Aerosol Technik, Germany, type 1.108). OPC can reliably measure particles in the 400 nm to 10 μm size range. We set the scanning time to 4 min and took at least 4 scans during each measurement. The gas flow in the equipment was 1 l/min.

### Morphology studies

#### Scanning electron microscopy (SEM) investigation of ablated tablet surfaces

To better understand the particle formation mechanism during ablation, we investigated the laser treated tablet surfaces, the morphology of the laser irradiated areas and the backscattered particles nearby using scanning electron microscopy (Hitachi S-4700). Prior to imaging, the samples were sputter-coated with gold (Bio-Rad SC 502). SEM images were recorded at a magnification of 100 ×.

### Optical absorption investigation

#### Ellipsometry

The optical absorbance of the target material plays a key role in laser ablation. Since there is no relevant and reliable information about the optical absorbance ([α] = 1/nm) of the studied drugs in the 400–1100 nm wavelength range, we determined the relevant values from ellipsometric data. Ellipsometric measurements were performed at different points on the tablets using a Woollam M2000F rotating compensator ellipsometer. Data were collected at 75° angle of incidence, applying focusing optics. The ellipsometric data measured in the 250–1000 nm wavelength range could be directly transformed to complex refractive index values, from which the absorption coefficients were determined according to the α = 4*π*κ/λ expression.

### Fast photography of the ablation process

**Figure 2:**
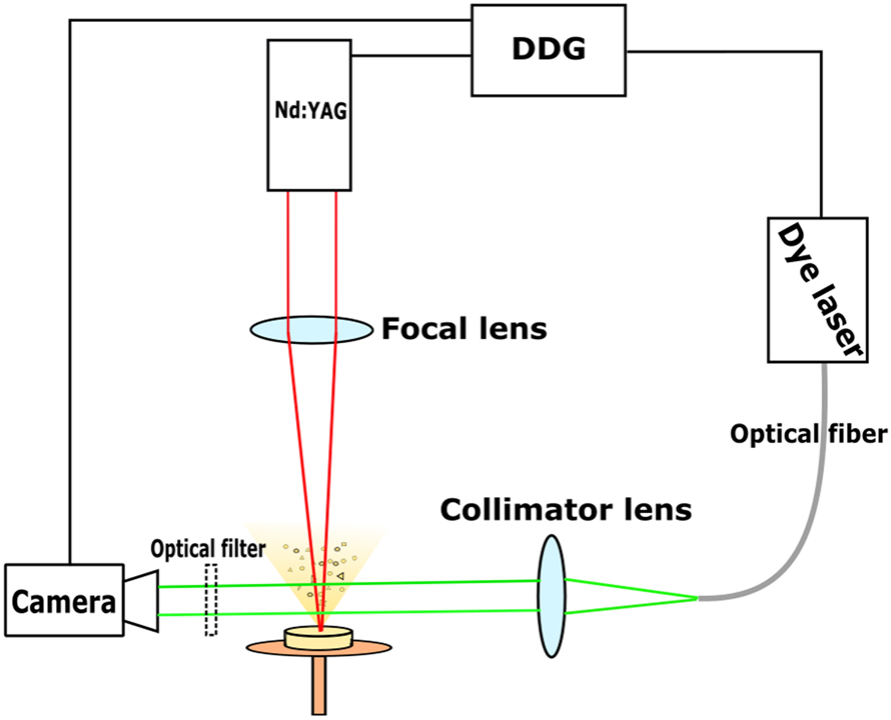
Pump and probe setup for fast photography.

We also set up a commonly used pump and probe system (Fig. 2) for the visual observation of the ablation process. The pump laser was the same Nd:YAG laser (Quantel, FWHM = 6 ns, λ = 532 nm/1064 nm) as for the ablation experiments. As probe lasers, we built two types of nitrogen laser induced dye lasers corresponding to the ablating wavelengths. In case of λ_ablating_ = 1064 nm we used Coumarin 153 as a laser active material in the dye laser (λ = 545 nm, FWHM = 1 ns), while in case of λ_ablating_ = 532 nm we had to change the laser active dye to Rhodamine 6G (λ = 590 nm, FWHM = 1 ns) in order to avoid an overlap between the pump and probe signals. The dye laser beams were transported to the experimental field by an optical fiber and collimated with an N-BK7 focusing lens (f = 15 cm). A CCD camera (The Imaging Source-DMK 23G445, 30 fps, N = 3 ×) was placed opposite the optical fiber. To filter out the white light of the generated plasma, we applied a bandpass optical filter in front of the camera. The central and controlling element of this setup was the digital delay generator (DDG) (SRS-DG645), which can separately trigger the pump and the probe lasers and the camera with a delay time in the nanosecond to millisecond range.

## Results

### Infrared spectroscopy (FTIR) of ablated drug particles

**Figure 3:**
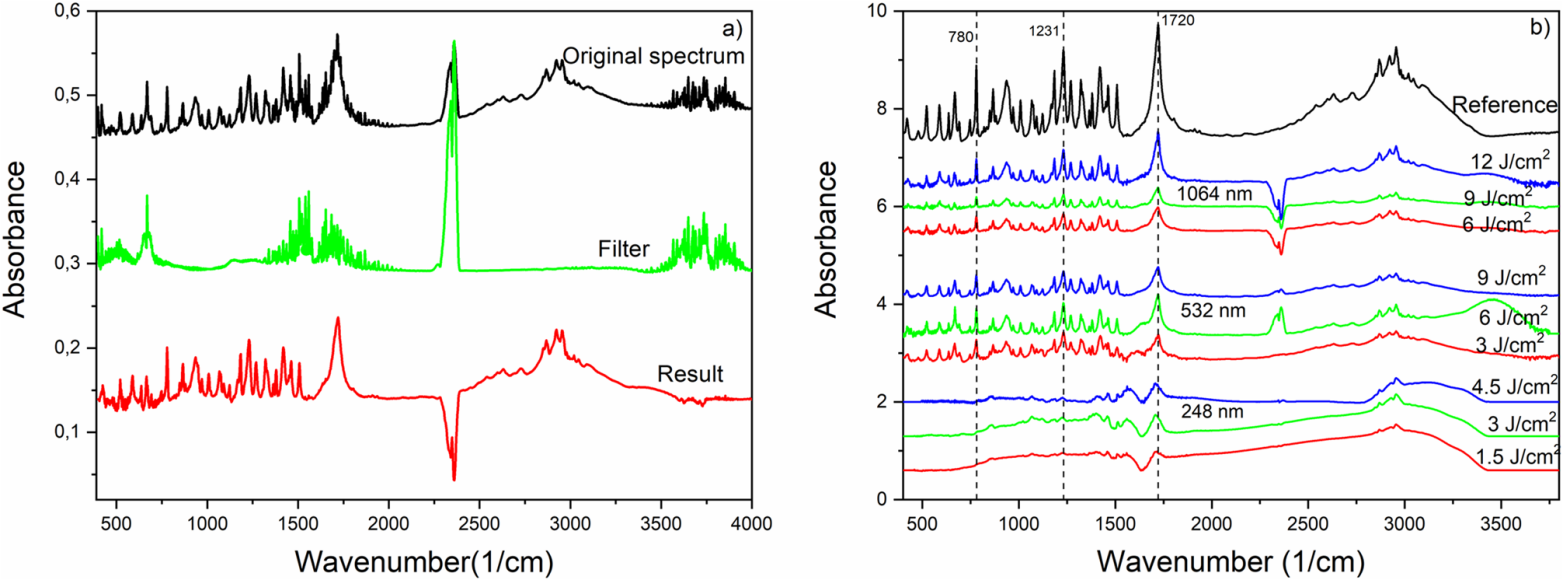
FTIR spectra of the produced ibuprofen particles; (**a**) measured spectrum of collected particles (black), background spectrum of filter (green), background-subtracted spectrum of particles (red); (**b**) background-subtracted FTIR spectra of ibuprofen particles prepared at different wavelengths and fluences.

FTIR spectra obtained from the collected particles are shown in Fig. 3a. Some contamination of the sample was inevitable while the collected particles were scraped or peeled off the filter. Therefore, we subtracted the FTIR spectrum of pure Millipore filter as background spectrum. The two peaks between 2250 and 2500 cm^−1^ are related to the H_2_O and CO_2_ content of the samples and give no relevant information for this investigation.

**Figure 4:**
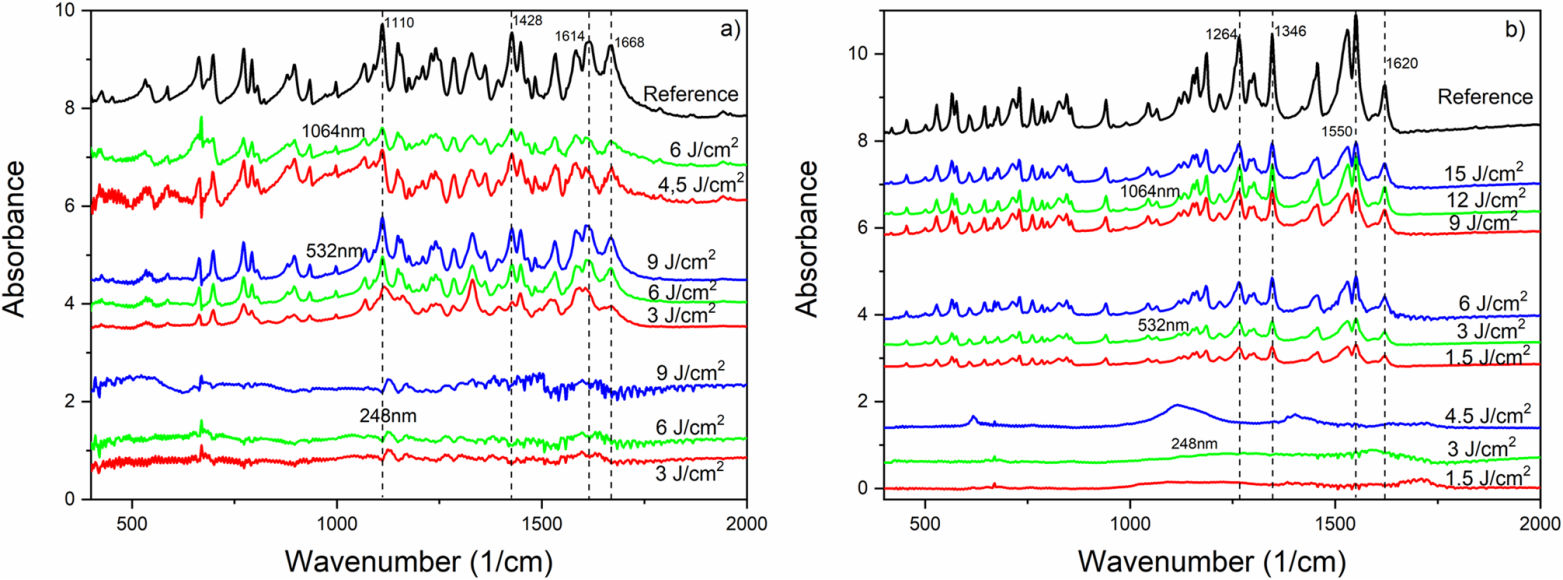
FTIR spectra of drug particles produced by laser ablation at different wavelengths and laser fluences; (**a**) niflumic acid; (**b**) meloxicam.

FTIR spectra of ibuprofen particles generated at different wavelengths and fluences are shown in Fig. 3. It can be seen that all laser fluences applied at the UV wavelength (248 nm) caused significant chemical damage to the particles. However, the persisting characteristic peaks of ibuprofen [e.g. 1720 cm^−1^ (H-bonded C=O); 1231 cm^−1^ (H-bonded CO–H); 780 cm^−1^ (C=O)]^26^ in the FTIR spectra indicate that no chemical changes occurred at VIS (532 nm) and IR (1064 nm) wavelengths. We had similar observations in the case of niflumic acid [e.g. 1668 cm^−1^ (C–O); 1614 cm^−1^ (benzene ring); 1428 cm^−1^ (C=O/OH); 1110 cm^−1^ (C–F)]^27,28^ and meloxicam [e.g. 1620 cm^−1^ (N–H); 1550 cm^−1^ (thiazole ring); 1346 and 1264 cm^−1^ (sulfone)]^29,30^ (Fig. 4). During IR ablation of niflumic acid, 4.5 J cm^−2^ and 6 J cm^−2^ laser fluences yielded a sufficient amount of particles for FTIR analysis.

Since we wanted to avoid chemical modification of the pharmaceutical compounds, we used only VIS and IR wavelengths for further investigations.

### Raman spectroscopy of generated drug particles

**Figure 5:**
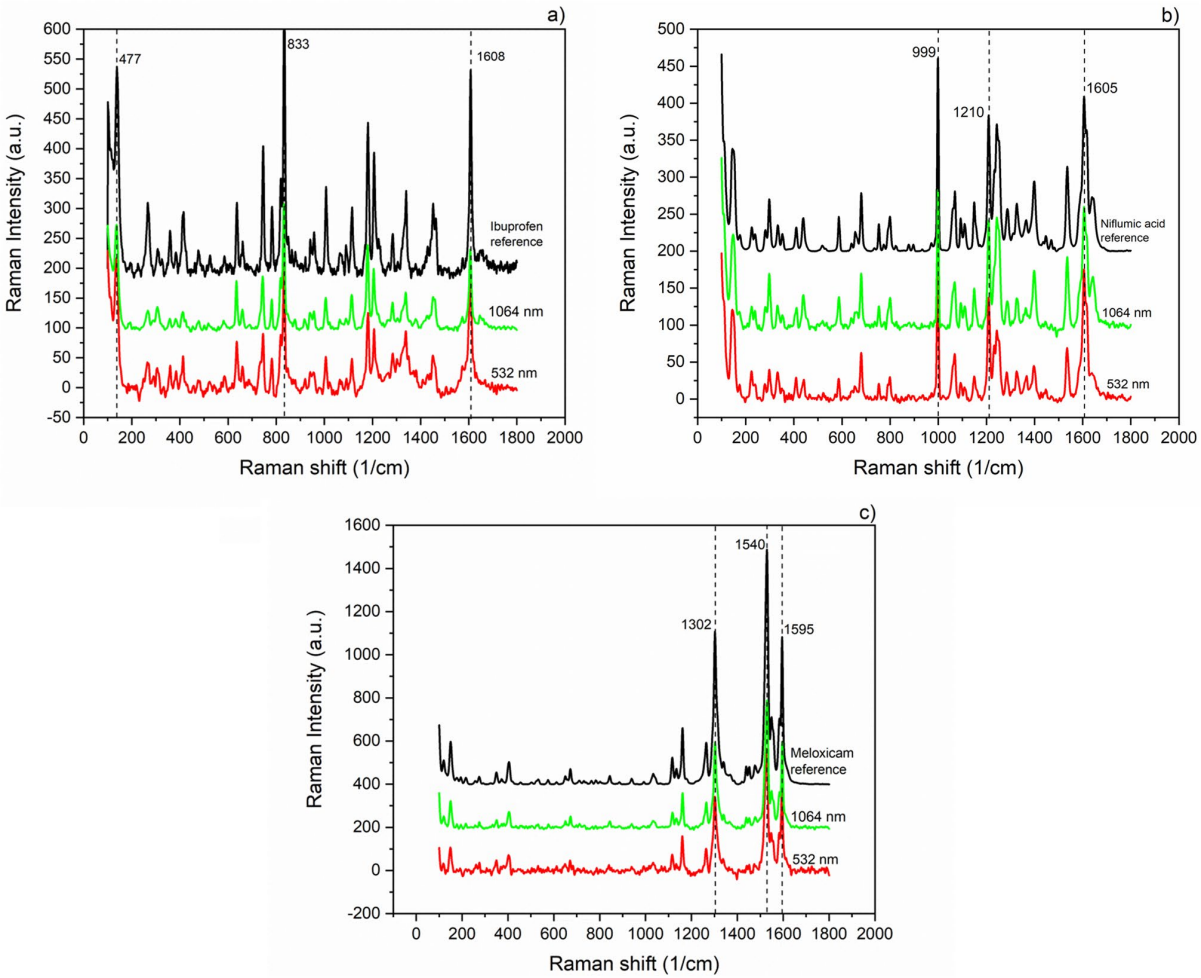
Raman spectra of drug particles produced by laser ablation at different wavelengths and at the highest applied laser fluences; (**a**) ibuprofen; (**b**) niflumic acid; (**c**) meloxicam.

We recorded several spectra at different places from each ablation product to obtain information about their homogeneity. For simplicity, in Fig. 5, only those spectra are compared with the original pharmaceuticals’ spectra that were recorded from the particles ablated by the highest laser fluences (i.e., may have suffered the most severe chemical changes) at both wavelengths. We marked some characteristic bands of ibuprofen (477, 833 and 1608 cm^−1^) (Fig. 5a), niflumic acid (999, 1210 and 1605 cm^−1^) (Fig. 5b) and meloxicam (1302, 1540 and 1595 cm^−1^) (Fig. 5c), and found no discrepancies in the spectra of the original drugs and their ablation products produced at 532 and 1064 nm.

### Particle size analysis

Using both SMPS and OPC, we investigated the size distribution of the ablated particles in a very wide range, from 10 nm to 10 μm. For each fluence, the repetition rates of the laser shots were adjusted to prevent the suction tube from getting clogged by ablated particles. During evaluation, we normalized the particle numbers to 1 Hz repetition rate for data comparison.

**Figure 6:**
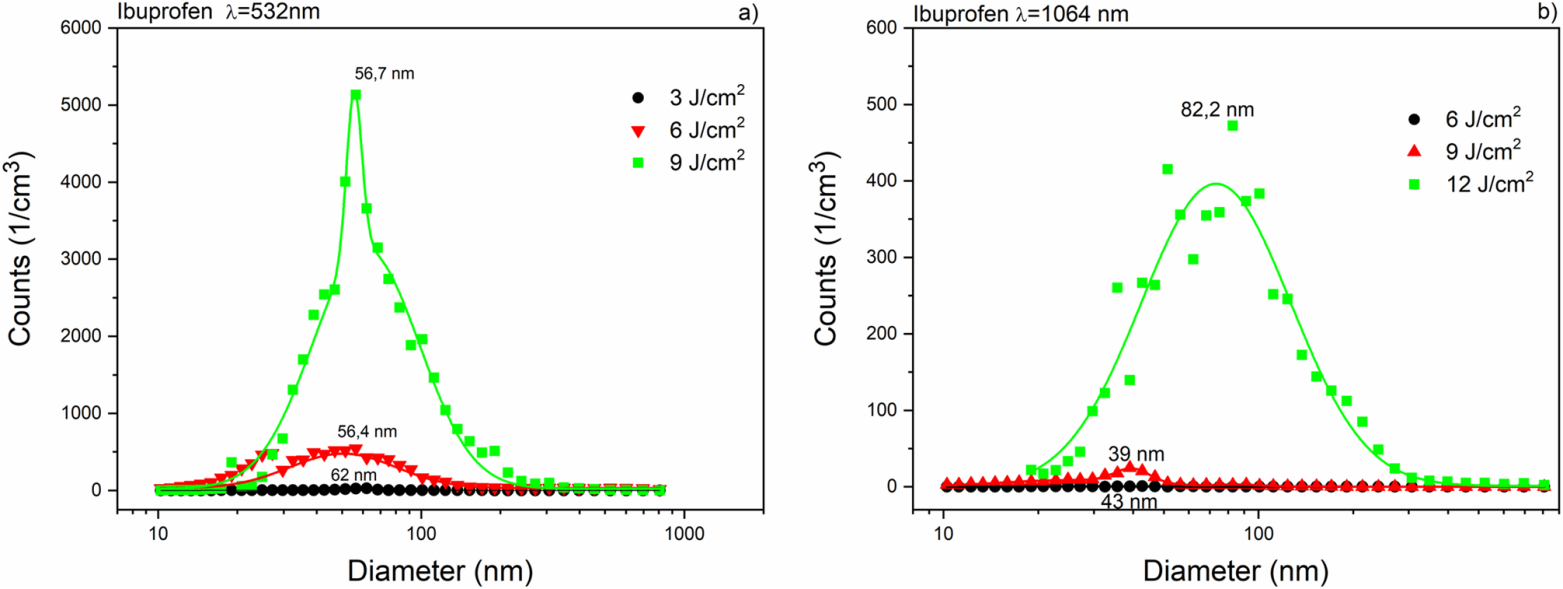
Size distribution of ibuprofen particles produced by laser ablation (**a**) at λ = 532 nm with 3 J cm^−2^ (black), 6 J cm^−2^ (red) and 9 J cm^−2^ (green) fluences; (**b**) at λ = 1064 nm with 6 J cm^−2^ (black), 9 J cm^−2^ (red) and 12 J cm^−2^ (green) fluences.

**Figure 7:**
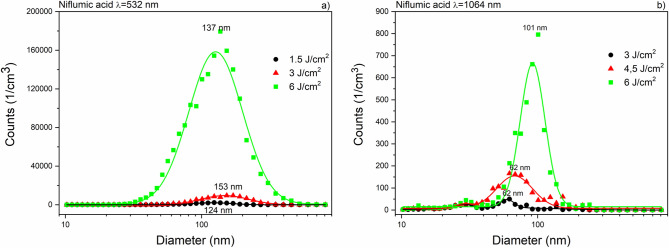
Size distribution of niflumic acid particles produced by laser ablation (**a**) at λ = 532 nm with 1.5 J cm^−2^ (black), 3 J cm^−2^ (red) and 6 J cm^−2^ (green) fluences; (**b**) at λ = 1064 nm with 3 J cm^−2^ (black), 4.5 J cm^−2^ (red), 6 J cm^−2^ (green) fluences.

**Figure 8:**
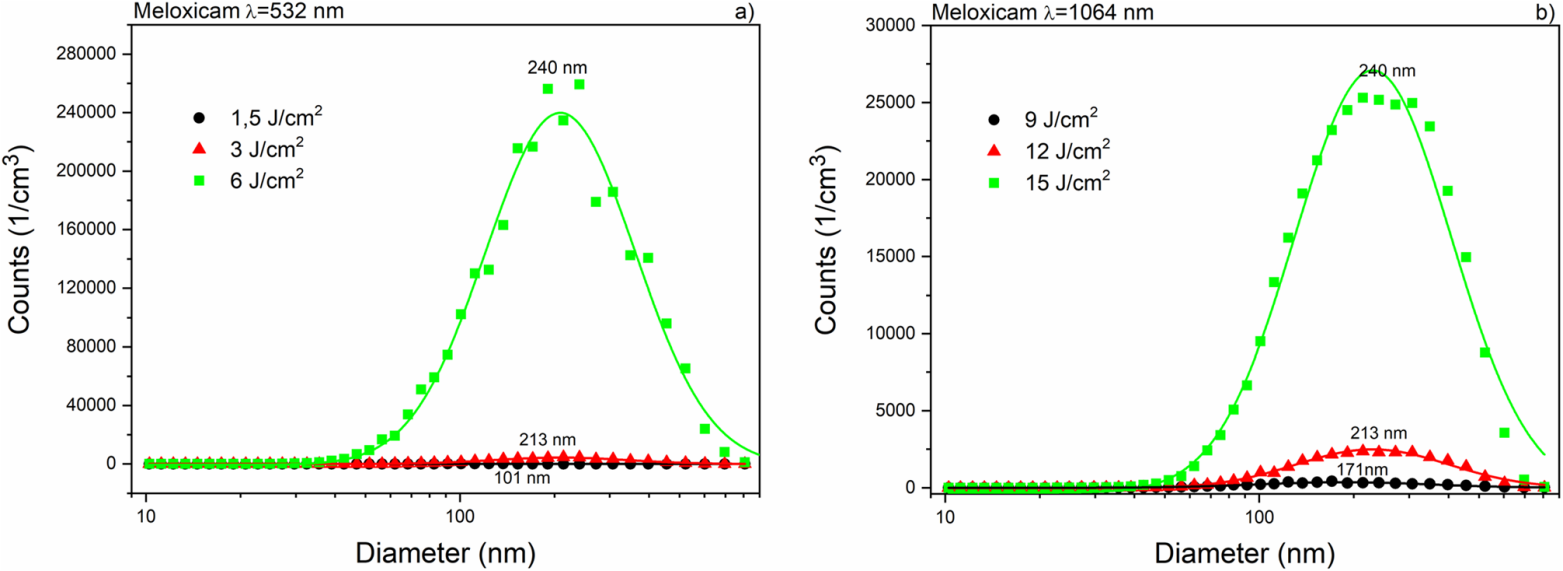
Size distribution of meloxicam particles produced by laser ablation (**a**) at λ = 532 nm with 1.5 J cm^−2^ (black), 3 J cm^−2^ (red) and 6 J cm^−2^ (green); (**b**) at λ = 1064 nm with 9 J cm^−2^ (black), 12 J cm^−2^ (red) and 15 J cm^−2^ (green).

Size distribution (measured by SMPS) of ibuprofen particles produced by laser ablation at 532 nm and 1064 nm wavelengths can be seen in Fig. 6. The mode values in both distributions were below 100 nm. The ablation yield of niflumic acid was considerably higher than for ibuprofen, especially at 532 nm (Fig. 7). The broadest size distributions with the highest mode values were obtained for the ablated meloxicam particles (Fig. 8); the full widths at half maximums (FWHMs) of the distributions ranged from 300 to 400 nm and the mode values for higher fluences exceeded 200 nm. We fitted one or more lognormal curves to the measurements. In case of high ablation yields data could be fitted to a single curve, while we had to cumulate two or more curves for small amounts of ablated particles.

**Figure 9:**
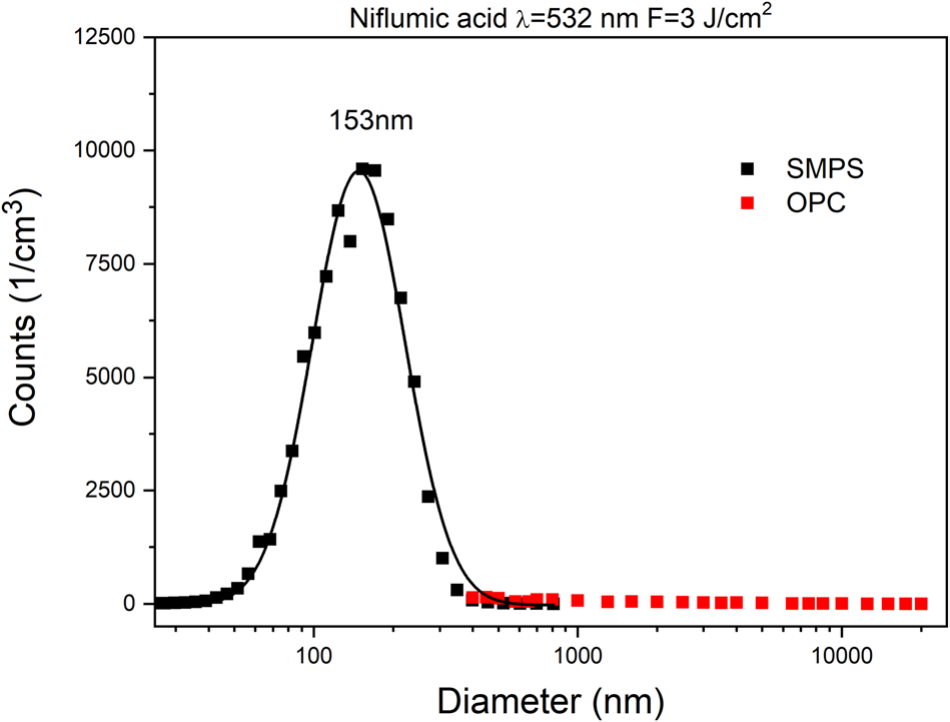
Size distributions obtained by SMPS and OPC for the same ablation parameters. The plots demonstrate a common overlap of the distributions.

All measurements above were also repeated with the OPC system to see if particles larger than 800 nm were also produced. Since OPC uses a higher gas flow than SMPS, the number of particles had to be normalized to the flow rate of SMPS and 1 Hz repetition rate. It must be noted that the two systems give different size values due to the different detection mechanisms (SMPS—by electrical mobility; OPC—by optical scattering). Hence the differences between them are minor and the values are mainly the same^31^. We did not find many particles larger than 800 nm in either case. Figure 9 shows the particle size distributions obtained by both SMPS and OPC for the ablation of niflumic acid. We chose this plot to demonstrate a common overlap of the distributions. The overlaps are the same for the other ablating parameters too.

The main experimental results are summarized in Table 1. There is no sign of clear relationship between laser fluences and size distributions in the investigated fluence range. However, we can establish that the number of ablated drug particles grows with the applied fluence at any wavelength. The growth in ablated particles can clearly be seen at λ = 532 nm ablation. In case of ibuprofen and niflumic acid, the most abundant particles are smaller at IR ablation than at VIS with equivalent fluences. In the case of meloxicam, there were no overlaps in the fluence values due to different ablating outcomes (thresholds, cracks). Nevertheless, we can still claim that at higher laser fluences the modes of meloxicam particle size distributions belong to the same size range for IR and VIS wavelengths. It can be clearly stated that in any case the efficiency of ablation (i.e. the number of ablated particles) is the highest for meloxicam, slightly lower for niflumic acid, while it is the lowest for ibuprofen.

**Table 1:** General information on the size distribution of ablated drug particles.

F (J cm^−2^)	D (nm)	FWHM (nm)	N_max_	F (J cm^−2^)	D (nm)	FWHM (nm)	N_max_
**Ibuprofen 532 nm**				**Ibuprofen 1064 nm**			
3	62	17	281	6	42.75	12	0.61
6	56.4	61	545	9	39	16	25
9	56.7	33	5131	12	82.2	84	472
**Niflumic acid 532 nm**				**Niflumic acid 1064 nm**			
1.5	123.71	97	2218	3	62	17.6	50
3	153	151	9590	6	62	45	166
6	137	123	179,400	9	101	37	795
**Meloxicam 532 nm**				**Meloxicam 1064 nm**			
1.5	100.89	101	115	9	170.49	297	414
3	213	234	4739	12	213	362	2374
6	240	300	259,158	15	240	372	25,306

*F* fluence, *d* diameter, *FWHM* full width at half maximum, *Nmax* number of particles at peaks of distribution.

### SEM investigation of ablated surfaces

**Figure 10:**
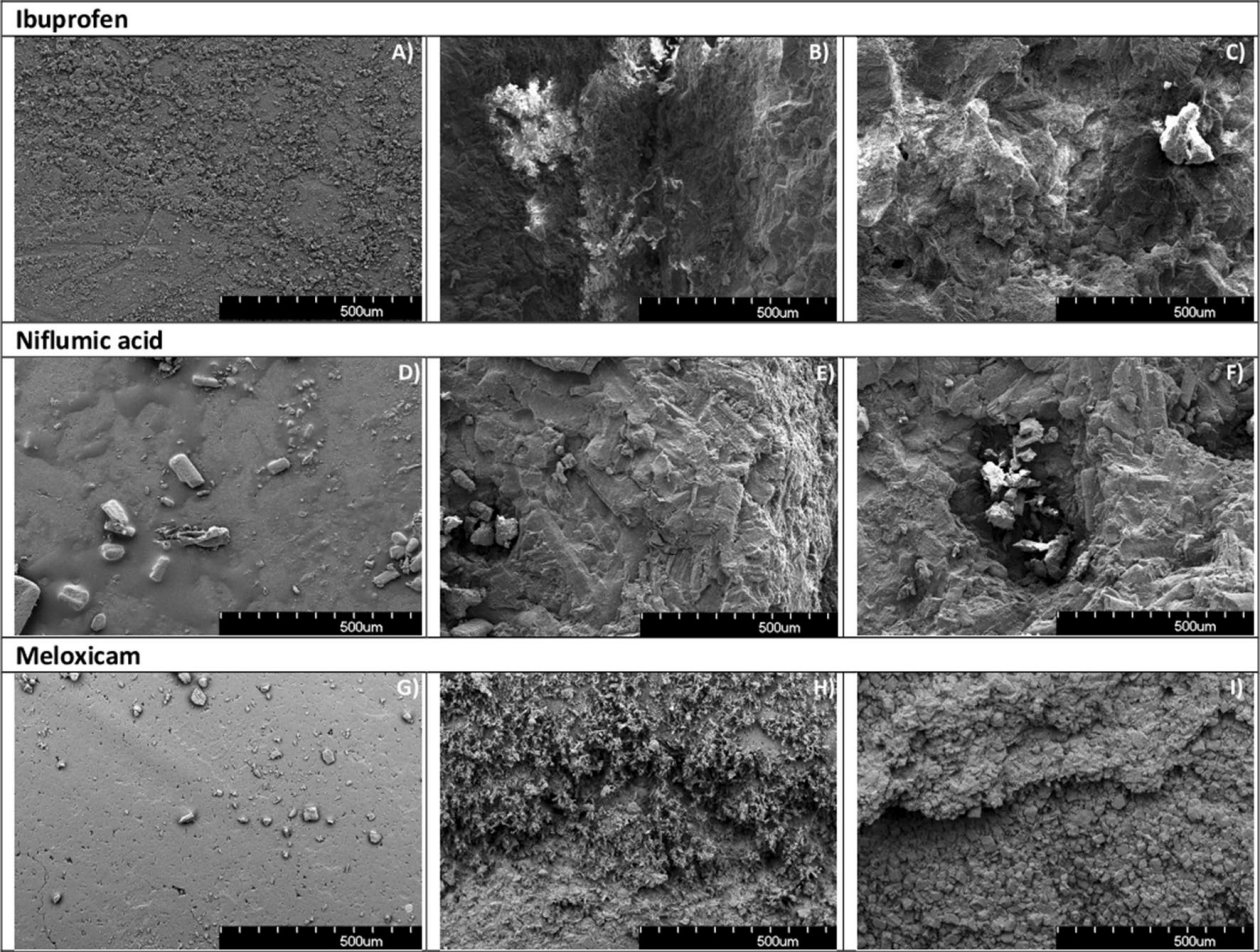
SEM images of intact and ablated surfaces of drug tablets; (**A**) intact ibuprofen surface, (**B**) ablated at 532 nm, (**C**) ablated at 1064 nm, (**D**) intact niflumic acid surface, (**E**) ablated at 532 nm, (**F**) ablated at 1064 nm, (**G**) intact meloxicam surface, (**H**) ablated at 532 nm, (**I**) ablated at 1064 nm.

SEM images taken from the intact and ablated drug tablet surfaces are shown in Fig. 10. Laser irradiations at VIS and IR wavelengths resulted in similar surface structures: crystal layers with sharp edges and cracks prevailed, and no smooth or melted areas could be found. This supports the observation that unmodified drug molecules can be produced by the PLA method.

### Ellipsometry

**Figure 11:**
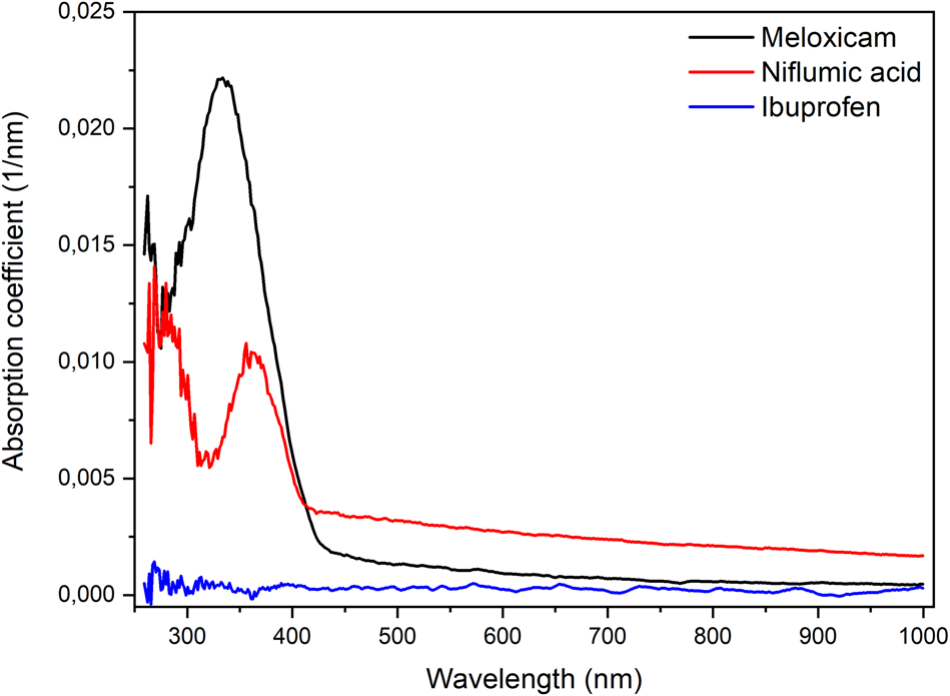
Absorption coefficients of meloxicam (black), niflumic acid (red), and ibuprofen (blue).

Absorption spectra of the investigated drugs were recorded in a wide optical range, from ultraviolet to near infrared. The smallest absorption coefficients were obtained in the IR, while the biggest ones in the UV range (Fig. 11). The increased absorption in the UV is most noticeable for meloxicam and niflumic acid. This is in accordance with our observation that drug molecules degrade during ablation by UV light. Optical absorption of ibuprofen has only been observed at around 250 nm^32^. This wavelength falls outside our measurement range (260–1000 nm), therefore we could not detect it.

We estimated the laser induced temperature rise at different depths in the target drugs by simple model calculations in which we varied the laser wavelength and fluence for each drug. Ignoring heat conduction, we applied the Beer–Lambert law (Eq. 1) to calculate the temperature change at the surface and at the penetration depths:

1 $$\Delta T(x) = \frac{\alpha *F}{{\rho *c}}*e^{ - \alpha x}$$

where α is the wavelength dependent absorption coefficient, *x* is the distance from the surface, *F* is the applied fluence of the laser beam, ρ is the density and *c* is the specific heat of the target material. We used the mean specific heat values between room temperature and the decomposition temperatures of the drugs in the absence of reliable data in the literature and since *c* is temperature dependent. We also calculated the reflexivity of the drugs for different incident angles and polarizations. Since reflexivity was under 10% for all wavelengths, we neglected it in the calculations. The parameters and the calculated penetration depths (d = 1/α) are summarized in Table 2. In all three medicines the highest temperatures occur at the UV wavelength. The temperature values are close at VIS and IR wavelengths, hence the differences between the absorption coefficient values are minor. At these two wavelengths, during ibuprofen ablation the estimated temperatures (depending on the fluences) exceed several thousand kelvins (2000–10,000 K) at the surface where the temperature values are the highest. This is followed in magnitude by meloxicam, where these values are higher and reach 10,000–20,000 K. Surface temperatures are the highest in niflumic acid, where these values reach 30,000–100,000 K. The temperatures at the laser penetration depths always significantly exceed the decomposition temperatures of the materials. Therefore, chemically preserved particles cannot originate from the volume element (V_e_)—defined by the laser spot size and penetration depth (V_e_ = A_spot_*d)—and these particles must be produced by another mechanism.

**Table 2:** Input data for temperature calculations; d (1/α)-optical penetration depth.

	ρ (kg*m^−3^)	c (J/(K*kg))	λ = 260 nm	λ = 532 nm	λ = 1000 nm	λ = 260 nm	λ = 532 nm	λ = 1000 nm
			α (1/μm)	α (1/μm)	α (1/μm)	d (nm)	d (nm)	d (nm)
Ibuprofen	1030	1553	0.514	0.2	0.305	1940	5000	3280
Nifl. acid	1400	2000	10	3	1.6	100	333	625
Meloxicam	1614	1800	14	1	0.407	71.42	1000	2146

### Fast photography

**Figure 12:**
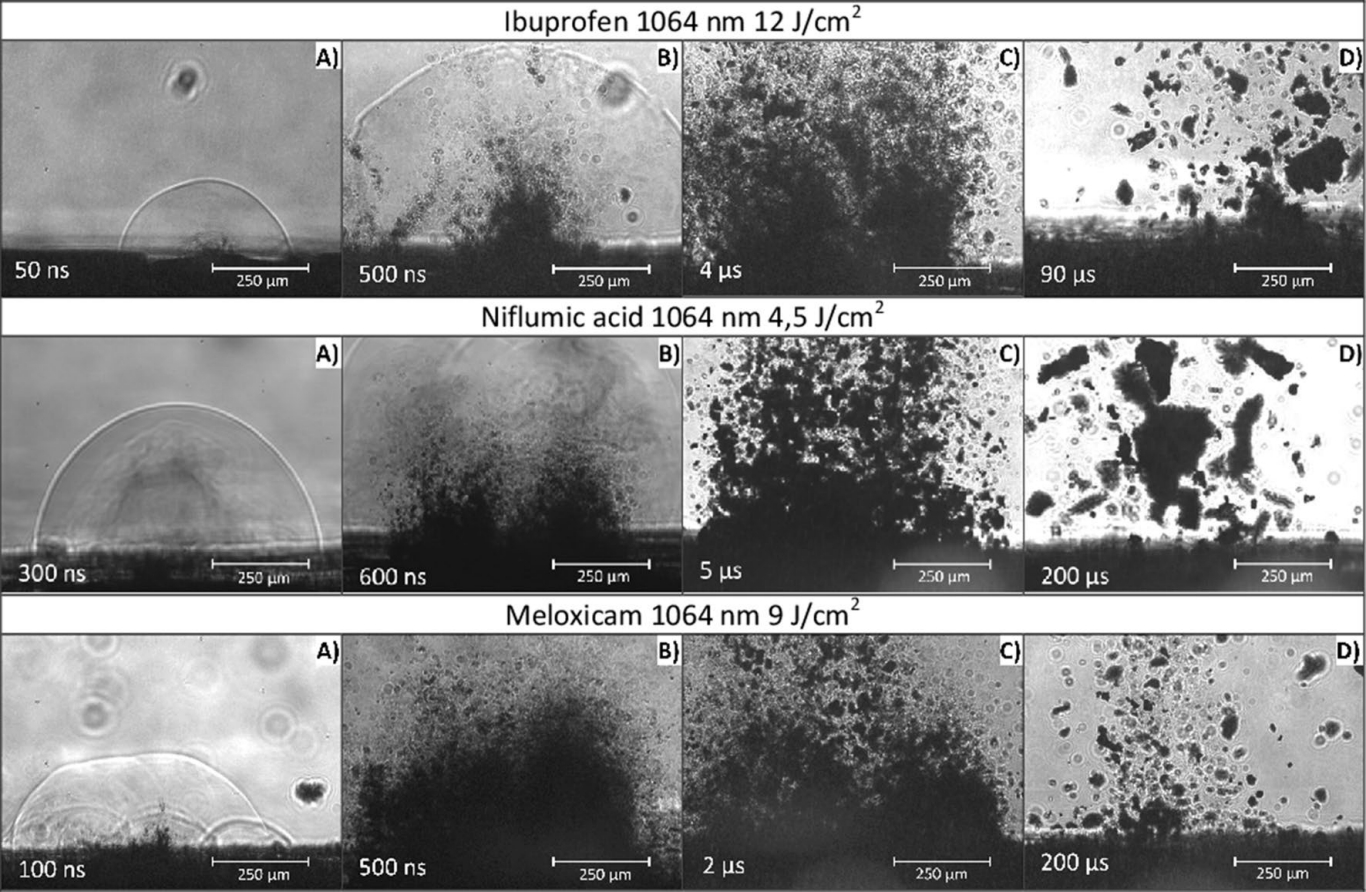
Fast photography images of drug ablations.

Successive stages of the ablation process were captured by fast photography. This information is valuable for us in representing and understanding the dynamics of particle generation. Figure 12 shows fast photography images deemed essential for the important stages of particle generation.

It can be clearly seen that the ablation mechanism is the same for all three studied drugs. The generated shock wave is closely followed by a cloud of non-condensed state of matter expanding at a slightly lower speed than the shock front. After that, solid particles increasing in size with time start to exit the surface, which means that bigger and heavier particles have lower velocities. It can be easily observed that particles of all sizes were generated during the ablation of drug tablets, although we could not detect a sufficient number of particles above 1 μm with the particle sizer methods. This can be explained with the mass dependence of the mean free path and the phenomenon that the magnitude of gas flow limits the sizes of transportable particles.

**Figure 13:**
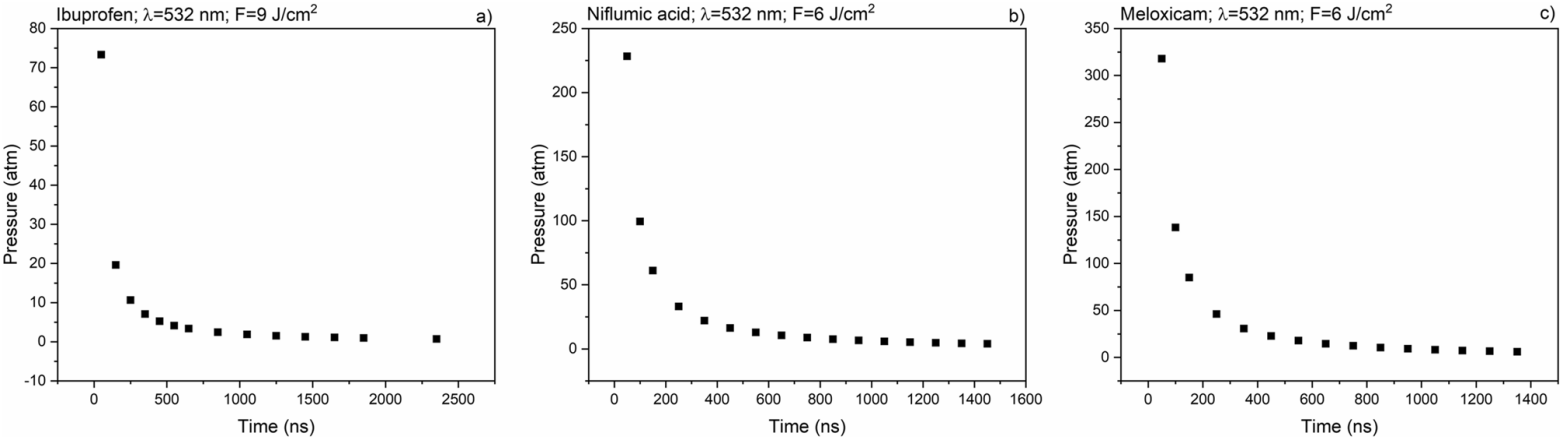
Pressure calculations for the shock fronts in case of (**a**) ibuprofen with 9 J cm^−2^; (**b**) niflumic acid with 6 J cm^−2^ and (**c**) meloxicam 6 J cm^−2^, λ = 532 nm.

To estimate the significance of photomechanical effects during ablation, we calculated the pressures produced in the target material at the shock fronts^33^. First we calculated the propagation velocity of the shock front by measuring its travel distance as a function of time, and then the pressure was estimated according to Eq. 2:

2 $$P = \frac{2}{\gamma + 1}*\rho *v^{2} ,$$

where P is the pressure produced at the shock front, ρ is the density of the medium, v is the velocity of the shock front and γ is the proportion of specific heats (c_p_/c_V_) of the medium. In Fig. 13 some examples of the calculated pressures are shown. The recoil pressures—which are equivalent with the initial pressures at the shock front—are 70–350 atm. These relatively high pressure values confirm our assumption, that the photomechanical effects have a significant contribution to the ablation process.

## Discussion

In the course of PLA, two successive and significantly different material ejection mechanisms can be distinguished: (I) Prompt evaporation of the target material directly from the center of the focused laser beam spot, where very high temperatures are created close to the surface. (II) Material ejection induced by the photomechanical effect due to pressure pulse and shock wave formation in the bulk material as a result of fast evaporation. According to our approximate calculations, temperatures as high as 10^3^ K to 10^4^ K can be created at the target surface. Some of the imparted energy is transferred inside the target material by heat conduction, and in the volume element (V_e_) the temperature exceeds a critical value and fast vaporization processes occur with the formation of high-pressure ionized plasma. In this nonequilibrium plasma micro-explosions take place, and the pressure pulse initiates the propagation of a high velocity shock wave outwards from the excited volume element. Then vaporized and highly decomposed products burst out as shown in Fig. 12A. However, a major part of the target material is ejected via photomechanical processes. The shock wave induced recoil forces and mechanical stresses result in the ejection of small and large fractures from the surface. The photomechanical ablation mechanism is more significant in case of nanosecond laser pulses^17^. The fractured particles leave the target at different velocities in accordance with their size and mass. This is in good agreement with our fast photography results (Fig. 12) where submicron sized particle ejection occurs at nanosecond and few microsecond time scales. Larger, micron-sized and irregularly shaped particles are ejected in the microsecond scale^16–19^.

The above observations apply mainly to homogeneous bulk materials. In case of drug tablets where the targets are highly porous and consist of micrometer-sized grains, the PLA process is slightly different. The uneven surface of the ablation holes, which can be clearly seen in the SEM images (Fig. 10) and the pressure calculations in indicate strong photomechanical effects. Thus, the subsequent laser pulses reach irregularly shaped target surfaces. The effect of target inhomogeneity is pictured in Fig. 12, as multiple shock waves are developing after one laser pulse. Consequently, the ideal homogenous optical absorption mechanism does not hold for our porous drug tablet targets, where lower temperature changes and photomechanical effects occur. Furthermore, since the drug grains are more loosely bound together in compacted tablets than in a bulk material, much higher ablation yields can be attained. Occasionally the recoil forces rip out large pieces (several microns in diameter) from the tablets. These phenomena explain the random fluence dependency in the size distribution of the ejected particles (Table 1). In bulk materials, the average size of the ablated particles and the FWHMs of the particles’ size distribution increase with the fluence^34,35^.

According to the FTIR investigations, PLA at UV (λ = 248 nm) laser wavelength is not an adequate method for creating chemically preserved particles. This conclusion is supported by the high absorption coefficients of drugs measured in the UV regime (see Fig. 11). Our calculations, which were based on the absorption coefficients, show that the UV laser beam creates the highest temperature in the excited volume element, and it has the lowest penetration depth in the target material (see Table 2). Therefore, the secondary photomechanical effects, which can lead to the ejection of chemically non-degraded particles, are less significant. Moreover, the energy of the photons (E_p_(λ = 248 nm) = 5 eV) exceeds the energy of several chemical bonds in the drug molecules, enhancing the probability of direct photochemical reactions like photolysis or photo dissociation. We concluded that by PLA at λ = 248 nm, the ejected particles consist mainly of degraded drug molecules, similarly to polymers seen before^36–38^.

At higher wavelengths, i.e. at lower photon energies [E_p_(λ = 532 nm) = 2.33 eV; E_p_(λ = 1064 nm) = 1.16 eV], the photochemical effects are less significant, but at the same time more drastic photomechanical effects can be expected. Due to the lower temperatures induced in the target material and the larger penetration depths of the VIS/IR laser beams, the secondary material ejection mechanisms are more prominent at 532 nm and 1064 nm laser wavelengths. This allows for the production of chemically preserved drug particles^39^. With the use of a laser beam at 532 nm considerably higher ablation yields could be achieved even at lower fluences than at 1064 nm (Table 1). This can be related to the higher (but still moderate) absorption coefficient at 532 nm. According to our observations, at 1064 nm laser wavelength the mechanical degradation (photo disruption) of the tablets was so severe that the tablets rapidly broke into pieces, greatly decreasing the ablation lifetime and ablation yield. A more elaborate evaluation of the ablation yields requires the consideration of the thermal and mechanical characteristics of the target materials too. It has been shown that there is a strong relationship between the mechanical properties (density, mechanical hardness) of the target and the ablation yield^40^. We suspect that the mechanical properties of the drug tablets influenced our experiments too. This can explain our observation that the ablation yields are higher for meloxicam than for niflumic acid although meloxicam’s absorption coefficients are lower at both wavelengths.

We must make two additional remarks. One concerns the absence of absorption bands in the FTIR spectra, which can be related to the degraded molecules. We suppose that at temperatures of thousands of kelvins in the evaporation zone the material is practically atomized/ionized, and therefore cannot be filtered out from the gas jet. This means that if there are any chemically modified but not completely atomized molecules, they must represent a negligible proportion of the whole ablation mass. Our other comment is that during the size distribution measurements (Fig. 9) no particles above ~ 1 μm in diameter were detected, although the formation of bigger particles can evidently be seen in the fast photography pictures (Fig. 12). Most probably, these bigger and heavier particles simply fell back to the surface, since even the highest adjustable gas flow was unable to transport them.

## Conclusion

PLA can be applied for the size reduction of poorly water-soluble NSAIDs. In the case of meloxicam, ibuprofen and niflumic acid, submicron to nanometer size particles can be produced by careful selection of the laser parameters, reducing the initial mean average sizes by orders of magnitude.

We have found that ablation without any chemical destruction of the initial pharmaceutical compounds can only be performed with laser beams having wavelengths in the VIS and IR range. Higher ablation yields are obtained at 532 nm than at 1064 nm laser wavelength, and the yields are generally increasing with the applied fluence for the studied drugs. Without aiming to conduct a thorough investigation we could also establish the influence of the absorption coefficients and the structure of the target drug tablets on the ablation mechanism.

The SMPS/OPC measurements provided particle size data precisely from that fraction of the laser ablated aerosol which can be pumped through a nasal delivery device and inhaled by the patient.

We concluded that PLA offers a chemical-free, verifiably reproducible and simple method for the size reduction of poorly water-soluble drug crystallites. The achieved submicron size range can be especially advantageous for the development of new pulmonary drug formulations for the medication of the lower lung area in pneumonia or severe acute respiratory syndrome. The adjustable laser parameters and thereby the fine tuning of the grinding mechanism during PLA can be advantageous over other commonly used methods when formulating drug delivery systems for sensitive compounds.
